# Diagnostic performance of host protein signatures as a triage test for active pulmonary TB

**DOI:** 10.1128/jcm.00264-23

**Published:** 2023-09-19

**Authors:** Lisa Koeppel, Claudia M. Denkinger, Romain Wyss, Tobias Broger, Novel N. Chegou, Jill M. Dunty, Kerry Scott, Tatiana Cáceres, Elloise Dutoit, Cesar Ugarte-Gil, Mark Nicol, Eduardo Gotuzzo, Paul L. A. M. Corstjens, Annemieke Geluk, Jayne Sutherland, George B. Sigal, Emmanuel Moreau, Audrey Albertini, Anna Mantsoki, Stefano Ongarello, Gerhard Walzl, Marta Fernandez Suarez

**Affiliations:** 1 Division of Infectious Disease and Tropical Medicine, University of Heidelberg, Heidelberg, Germany; 2 FIND, Geneva, Switzerland; 3 German Center for Infection Research (DZIF), Heidelberg University Hospital Partner Site, Heidelberg, Germany; 4 DSI-NRF Centre of Excellence for Biomedical Tuberculosis Research, South African Medical Research Council Centre for Tuberculosis Research, Division of Molecular Biology and Human Genetics, Faculty of Medicine and Health Sciences, Stellenbosch University, Cape Town, South Africa; 5 Meso Scale Diagnostics, LLC, Rockville, Maryland, USA; 6 Instituto de Medicina Tropical Alexander von Humboldt, Universidad Peruana Cayetano Heredia, Lima, Peru; 7 Division of Medical Microbiology at the University of Cape Town (UCT), Cape Town, South Africa; 8 School of Medicine, Universidad Peruana Cayetano Heredia (UPCH), Lima, Peru; 9 Division of Infection and Immunity, School of Biomedical Sciences, University of Western Australia, Perth, Australia; 10 Department of Cell and Chemical Biology, Leiden University Medical Center, Leiden, the Netherlands; 11 Department of Infectious Diseases, Leiden University Medical Center, Leiden, the Netherlands; 12 TB Research Group, Vaccines and Immunity Theme, MRC Unit The Gambia at LSHTM, Banjul, Gambia; University of Manitoba, Winnipeg, Manitoba, Canada

**Keywords:** *Mycobacterium tuberculosis*, host marker, biomarker, machine learning, diagnostics

## Abstract

The current four-symptom screen recommended by the World Health Organization (WHO) is widely used as screen to initiate diagnostic testing for active pulmonary tuberculosis (TB), yet the performance is poor especially when TB prevalence is low. In contrast, more sensitive molecular tests are less suitable for placement at primary care level in low-resource settings. In order to meet the WHO End TB targets, new diagnostic approaches are urgently needed to find the missing undiagnosed cases. Proteomics-derived blood host biomarkers have been explored because protein detection technologies are suitable for the point-of-care setting and could meet cost targets. This study aimed to find a biomarker signature that fulfills WHO’s target product profile (TPP) for a TB screening. Twelve blood-based protein biomarkers from three sample populations (Vietnam, Peru, and South Africa) were analyzed individually and in combinations via advanced statistical methods and machine learning algorithms. The combination of I-309, SYWC and kallistatin showed the most promising results to discern active TB throughout the data sets meeting the TPP for a triage test in adults from two countries (Peru and South Africa). The top-performing individual markers identified at the global level (I-309 and SYWC) were also among the best-performing markers at country level in South Africa and Vietnam. This analysis clearly shows that a host protein biomarker assay is feasible in adults for certain geographical regions based on one or two biomarkers with a performance that meets minimal WHO TPP criteria.

## INTRODUCTION

New diagnostic approaches are urgently needed to find the “missing millions” of tuberculosis (TB) patients who are neither diagnosed nor reported and to meet WHO End TB targets (1). Molecular tests such as Xpert MTB/RIF (Cepheid) (hereafter referred to as “Xpert”) or Truenat MTB-RIF Dx (Molbio) are more sensitive than smear microscopy, faster than traditional culture methods and have been rolled out around the world (2). However, placement of these instruments at primary care level in low-resource settings, where the majority of individuals first present for care, is restricted by infrastructure requirements and high cost (3, 4).

Currently, the WHO-recommended four-symptom screen (W4SS: presence of either current cough, fever, night sweats, or weight loss) is widely used as a screen to initiate diagnostic testing for pulmonary TB (PTB). However, the performance of such a screen is poor especially when TB prevalence is low, and better solutions are needed (5). A two-stage diagnostic algorithm in which a highly sensitive and moderately specific test, a so-called “triage- or rule-out test,” applied first at peripheral healthcare settings, could be a cost-effective approach to improving diagnostic yield. Such a test would effectively capture most subjects at higher likelihood of having TB and thus decrease the cost of the expensive confirmatory testing. The target product profile (TPP) suggested by the World Health Organization (WHO) for such a triage test specifies a minimum sensitivity of 90% and specificity of 70% relative to the confirmatory test, for identifying active TB disease and discriminating against the absence of disease (with or without TB infection), or respiratory diseases other than TB (ORD) (6).

Proteomics-derived blood host biomarkers have been explored to develop novel diagnostics for TB (7, 8). They are particularly attractive as protein detection technologies suitable for the point-of-care (POC) are well established, and could meet the cost targets put forward by WHO for a viable diagnostic (6). Current lateral flow technology can cover three biomarkers on one cartridge (9 – 11). Commercial examples of such multiplex antigen tests with and without a reader are the BD Veritor SARS-CoV-2 and Flu A + B combo test by Becton Dickinson and Company (12), Status COVID-19/FLU Test by Chembio (13), Sofia 2 Flu + SARS Antigen Fluorescent Immunoassay by Quidel (14) and multiplex immunoassays for myocardial infarction such as Quidel Triage Cardiac Panel by Quidel (15). Recently a novel lateral flow-based multi-biomarker test was reported for quantitative detection of six biomarkers, indicative for the humoral and cellular response upon infection with the *Mtb*-related *Mycobacterium leprae* (16).

An original large-scale proteomic discovery approach using the SOMAscan (SomaLogic, Boulder, CO, USA) discovered a combination of six biomarkers of high promise and identified additional markers with discriminatory power and large median fold-changes (8). Separately, an approach by Walzl et al. captured a partly overlapping, separate group of biomarkers that showed promise to be investigated further (17 – 20).

In this study, we describe the performance of 12 blood-based host protein biomarkers, selected based on these two prior workstreams, individually and in combination using advanced analysis methods and explore whether the required triage test performance can be met with a protein host-marker signature. We further establish concentration ranges, and nominate key protein marker candidates for translation into such a POC test.

## MATERIALS AND METHODS

### Study design and sample collection

Consecutive patients aged 18 years or older presenting with signs and symptoms of TB (cough for at least 2 weeks, fevers, weight loss, and night sweats) and able to provide informed consent were enrolled in tertiary referral centers in South Africa (Division of Medical Microbiology at the University of Cape Town), Peru (Instituto de Medicina Tropical Alexander von Humboldt at Universidad Peruana Cayetano Heredia, Lima), and Vietnam (ham Ngoc Thach Hospital, Ho Chi Minh City). Individuals with signs compatible with only extrapulmonary disease and those having received more than two doses of anti-TB therapy prior to enrolment were excluded. Basic demographic information was collected, such as age, weight, and gender, and clinical metadata, such as HIV status and, in some cases, CD4 cell counts and viral load. Data were captured through a dedicated, online, password-protected double data entry system. Chest radiographs were performed and interpreted by local radiologists in a subset of patients (Peru and Vietnam). Participants were asked to provide two spot sputum samples, serum, and plasma within 2 days for testing, and HIV testing was offered. The reporting of this study followed the STARD guidelines. All testing for the purpose of the reference standard was performed on fresh samples. All serum samples obtained at baseline for biomarker testing, were given a unique barcode, and frozen on-site in 0.5 mL aliquots prior to shipment to a central repository, where they were stored at −80°C prior to testing for this study.

### Biomarkers—index test

The biomarker candidates were selected in close discussion with investigators from earlier studies (17 – 20), considering and balancing individual discriminatory performances, median fold-changes, and feasibility of detecting the marker or markers in a POC test. From the original SOMAscan discovery work (8), kallistatin, SYWC (an interferon-γ inducible Trp-tRNA-synthetase), and complement component 9 (C9) were selected as they were the first markers when ranked by Kolmogorov-Smirnov (KS) statistic and were part of the original 6-marker signature. Serum amyloid A (SAA) and non-pancreatic Secretory Phospholipase A2 (NPS-PLA2) were included due to their large median fold-change and presence among the top-15 markers. From the work of Walzl et al. (19), ferritin, apolipoprotein A1 (ApoA1), CXCL10 or IP-10, CCL1 or I-309, and CXCL9 or MIG were selected. Finally, two previously described markers with diagnostic and treatment monitoring potential were included as point of comparison, namely lipopolysaccharide-binding protein (LBP) and C-reactive protein (CRP) (21). CRP is now endorsed by WHO for screening for TB (22) and was among the top 20 markers in the SOMAscan work which made up an optimal diagnostic bio-signature when combined with I-309 as shown by Walzl et al. In a screen of biomarkers correlating with treatment effect, LBP and CRP were among the top markers with the largest average decreases upon TB treatment (23).

### Reference standard and case definitions

Two sputum samples per study participant were obtained and each tested by acid-fast staining, liquid culture using mycobacteria growth indicator tubes with a BACTEC 960 instrument (BD Microbiology Systems, Sparks, MD, USA), solid culture with Löwenstein-Jensen medium, and where available, the XpertMTB/RIF [Cepheid, Sunnyvale, CA, USA (Xpert)] test. A culture-positive case was defined as a participant with at least one culture testing positive for *Mycobacterium tuberculosis* (MTB) in either of the two sputum samples in either solid and liquid culture. A culture-negative case was defined as a participant with all negative cultures for MTB (out of four; two solid and two liquid cultures) and negative cultures in follow-up. Study participants were considered smear-positive, if they had at least one positive smear with acid-fast staining. Participants with unclear microbiological diagnoses were excluded from the analysis, including those with missing culture results, those classified as smear-positive but culture-negative, and those only showing growth of non-tuberculous mycobacteria.

Patients were categorized based on clinical and microbiological results. Patients with positive MTB cultures were diagnosed as definite tuberculosis and subcategorized into smear-positive and smear-negative groups. Participants who were smear and culture negative but responded to empiric tuberculosis treatment were classified as “clinical tuberculosis” (CXR may be abnormal or not). Participants who were smear-negative, Xpert and culture negative on all sputum samples and who exhibited symptom resolution in the absence of tuberculosis treatment at the 2- to 3-month follow-up visit were classified as “non-tuberculosis disease.” See the Table S5.

### MSD U-PLEX assay testing

Frozen serum aliquots were sent to Meso Scale Discovery, LLC (MSD). Test operators at MSD were blinded to other test results. Custom immunoassay panels for the pre-defined host biomarkers were developed employing a multiplexed sandwich immunoassay format and electrochemiluminescence (ECL) detection and carried out on commercial instrumentation and multi-well plate consumables from MSD (24). The host biomarker panels were developed and optimized for multiplexing on the MSD U-PLEX assay platform, and capture antibody arrays were formed according to the manufacturer’s instructions.

The assay components for each panel included a 96-well plate having an array of capture antibodies in each well being monoclonal when possible (generated by the binding of capture antibodies labeled with U-PLEX linkers), a set of labeled detection antibodies (frequently polyclonal) for each analyte in the panel (labeled with the MSD SULFO-TAG ECL label), an assay diluent, a detection antibody diluent, a wash buffer, an ECL read buffer (MSD Gold Read Buffer A), a calibration standard containing a blend of the target analytes and a set of controls. Blended-analyte diluent-based controls were created for each panel at two concentration levels (high and low). A matrix-based control was also created by screening and pooling MSD-provided human serum samples. Each plate included the calibration standard, the set of controls and the serum samples analyzed in duplicate wells. Additional details are described in the Supplement Text S1.

To address the wide range of concentrations covered by the targeted biomarkers, literature research and pre-tests with a small number of samples at different dilutions were used to understand the concentration range per biomarker and to group biomarkers into three panels having different sample dilutions. Panel 1 (IP-10, I-309, and MIG) was run using a 1:2 sample dilution, Panel 2 (NPS-PLA2, Ferritin, and SYWC) was run using a 1:50 sample dilution, and Panel 3 (ApoA1, C9, CRP, kallistatin, LBP, and SAA) was run using a 1:50,000 sample dilution. Based on the use of CRP as a WHO-recommended screening test, the CRP result from Panel 3 is also presented independently as a comparator.

### Statistical analysis

We evaluated the diagnostic performance of 12 host biomarkers for diagnosing active TB by means of the efficient use of the machine learning algorithms (25) with the programming language python (Version 3.8) and the *scipy* library. The corresponding programming code and data have been made publicly available under references (26) and (27), respectively.

The statistical analysis was first performed on each of the biomarkers individually, then on all possible biomarker combinations. We compared the goodness of fit between the combinations by the value of their negative log-likelihood of the fitted binomial model accounting for country and human immunodeficiency virus (HIV) effect. This estimator is a transformation of the maximum likelihood value yet preserving numerical stability. It allows a ranking of the different combinations, because the smaller the negative log-likelihood value the better is the model fit. For each fixed number of biomarkers, we further analyzed the top three performing biomarker combinations, ranked by their negative loglikelihood value, by using a variety of methodologically varying supervised machine learning algorithms (28, 29): This includes logistic regression from a generalized linear model perspective, random forests representing an ensemble method, support vector machines representing a non-probabilistic binary linear classifier, and Naïve Bayes representing a probabilistic classifier.

The aim of this analysis was to investigate the potential of algorithms to discern between active TB disease and no TB disease, and to identify promising biomarker combinations for further research. As an exploratory analysis, the full data set was analyzed using fivefold cross-validation (alternating 80% of the data for training and 20% for testing) in order to make the best use of the limited data available. The algorithm produced a number between 0 and 1. A (shifting) percentage threshold determined whether an algorithmic outcome for an individual was denoted as having TB or no TB. Comparing the resulting classification with the reference standard in the data led to sensitivity [True positives/(True positives + False negatives)] and specificity [True negatives/(True negatives + False positives)] estimates. We did not aim at validating a certain algorithmic cutoff for one specific sensitivity and specificity value pair, which is why we did not set aside a separate part of the data set for this validation purpose.

We calculated receiver operator characteristic (ROC) curves for each selected biomarker combination and model and compared the results with the TPP goal suggested by the WHO of at least 90% sensitivity and 70% specificity against the reference standard (6).

To be able to compare biomarker combinations in their potential of ruling in the disease (high sensitivity), the respective sensitivity was calculated given the minimum TPP specificity target of 70%.

We further analyzed the algorithms’ performance on subsets by country and HIV status.

## RESULTS

### Study participants

**Fig 1:**
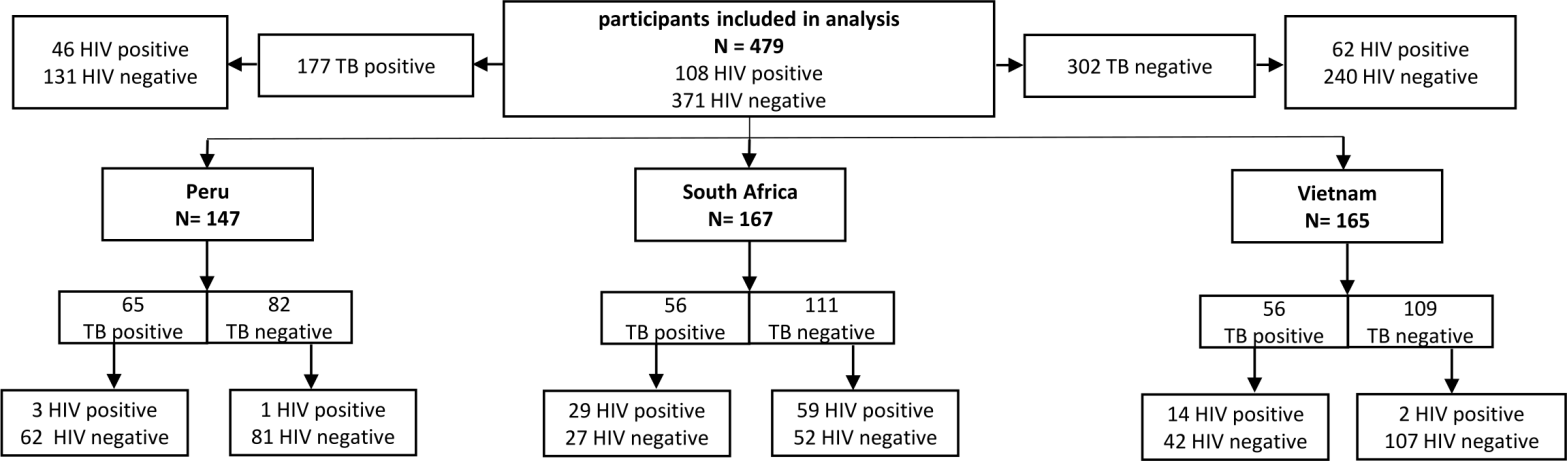
Stratification of the population into country, TB and HIV status.

A total of 479 adults were analyzed in a retrospective nested cohort design with 177 definite tuberculosis cases, and 302 non-tuberculosis diseases (Fig. 1). The data set recorded about the same number of individuals in the respective countries with different prevalence across the countries. In Peru, about half of the patients (44%) were TB positive, whereas the prevalence was lower in the other countries (34% in both). In South Africa, 53% of the patients were HIV positives regardless of TB status, whereas Peru (0.03%) and Vietnam (10%) showed lower numbers of HIV coinfection.

### Host biomarker diagnostic evaluation

**Fig 2:**
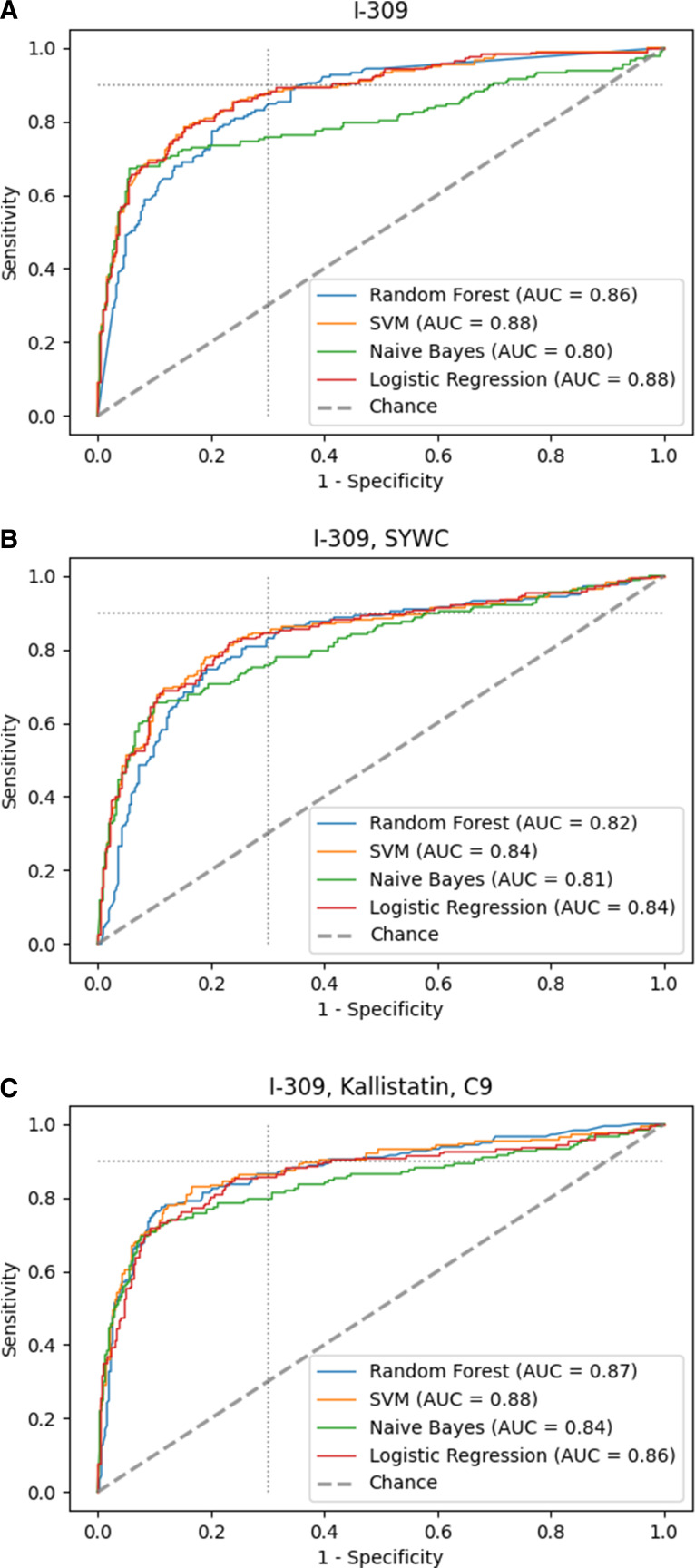
Receiver operator characteristic (ROC) curve of different machine learning algorithms with fivefold cross-validation for best performing (**A**) single biomarker, (**B**) combination of two, and (**C**) three different biomarkers. The dotted light gray line indicates the minimal TPP target with 90% sensitivity and 70% specificity. With the signature consisting of I-309, SYWC, and kallistatin the TPP could be reached by the random forest algorithm. AUC, area under the curve; SVM, support vector machine.

**Fig 3:**
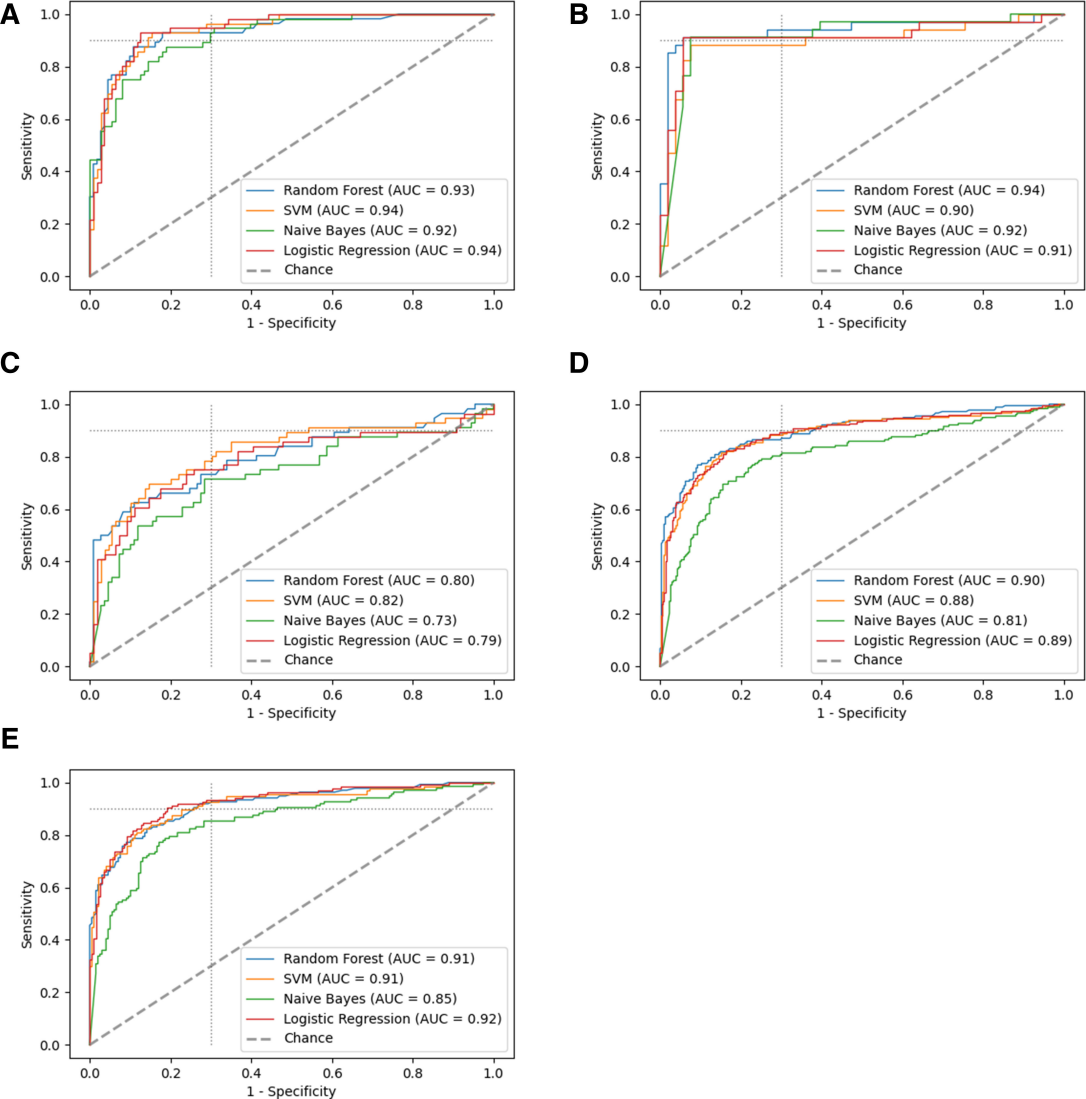
The ROC plot of all 12 biomarkers combined for (A) South Africa alone, (B) Peru alone, (C) Vietnam alone, (D) the whole data set, and (E) the whole data set excluding Vietnam. The dotted light gray line indicates the TPP with 90% sensitivity and 70% specificity. AUC, area under the curve; SVM, support vector machine.

Table 1 shows the biomarkers and biomarker combinations ranked by their negative log-likelihood value. For the top performing one, two, and three biomarker combinations from Table 1, and for the combination of all 12 biomarkers, Fig. 2 and 3 compare the ROC curves generated using four different algorithms, namely logistic regression, random forests, support vector machines, and Naïve Bayes. When analyzing single markers, I-309 was top performing according to the value of its negative log-likelihood [area under the curve (AUC) with logistic regression, 0.87], followed by SYWC and MIG (AUC 0.86 and 0.83, respectively). Quantitative information on the host markers’ concentration and fold-changes at global and regional levels can be found in Table S1 through S4 in the Appendix.

**TABLE 1:** Biomarker combinations ranked according to their value of the negative log-likelihood using the combined data from the three study sites

No. of biomarkers combined	Best combination [marker(s)] Log-likelihood, AUC for logistic regression	Second best combination [marker(s)] Log-likelihood, AUC for logistic regression	Third best combination [marker(s)] Log-likelihood, AUC for logistic regression
**1**	[I-309] 210, 0.87	[SYWC] 213, 0.86	[MIG] 244, 0.83
**2**	[I-309, SYWC] 189, 0.88	[I-309, kallistatin] 199, 0.87	[kallistatin, SYWC] 201, 0.88
**3**	[I-309, SYWC, kallistatin] 182, 0.90	[I-309, SYWC, ApoA1] 188, 0.89	[I-309, SYWC, NPSPLA2] 189, 0.89
**4**	[I-309, SYWC, kallistatin, C9] 179, 0.89	[I-309, SYWC, kallistatin, NPSPLA2] 180, 0.90	[I-309, SYWC, kallistatin, ApoA1] 181, 0.90
**5**	[I-309, SYWC, kallistatin, C9, LBP] 174, 0.90	[I-309, SYWC, kallistatin, SAA, C9] 175, 0.89	[I-309, SYWC, kallistatin, C9, NPSPLA2] 175, 0.90
**6**	[I-309, SYCW, kallistatin, C9, LBP, ApoA1] 174, 0.90	[I-309, SYWC, kallistatin, SAA, C9, LBP] 174, 0.89	[I-309, SYWC, kallistatin, C9, NPSPLA2, SAA] 174, 0.90
**12**	[NPSPLA2, SYWC, C9, LBP, CRP, SAA, kallistatin, ferritin, IP-10, I-309, MIG, ApoA1] 172, 0.89		

The logistic regression model combining I-309 with SYWC provided a small improvement in the performance relative to I-309 alone (AUC 0.88). The analysis of the ROC curves (Fig. 2) for the individual combinations revealed that adding kallistatin as a third marker enhanced the performance in all algorithms confirming the inference (AUC 0.9 for logistic regression compared to 0.87 for I-309 alone). While the Naïve Bayes approach performed the poorest (Fig. 2C), the triage TPP suggested by the WHO was reached by the random forest algorithm using the combination of I-309, SYWC, and kallistatin.

We observe a nested behavior of the top-performing biomarker subsets, meaning that the biomarker combinations with less biomarkers were contained in the top-performing compounds with more biomarkers. However, combining more than three biomarkers provided no further improvement in the algorithmic performance or the AUC values. For some models, we observed a slightly lowered AUC value, in particular when using the full data with 12 biomarkers (AUC decreases for logistic regression to 0.89 in Fig. 3D compared to Fig. 2C), which can be attributed to overfitting and the randomness of the data sample.

Interestingly, the biomarker ApoA1 had very poor performance on its own (AUC 0.5; see Appendix), though it seemed to have great additional effect as the second best combination in a subset with three biomarkers [(I-309, SYWC, and ApoA1), AUC 0.89].

Given the diagnostic performance in the ROC curve and the ranking among the other biomarkers in its group, the combination of I-309, SYWC, and kallistatin served as the most promising combination to discern TB status for the whole data set reaching the Triage TPP suggested by the WHO. However, this was reached with only one out of four algorithms applied. Figure 2 clearly shows that adding more biomarkers leads to more information for the algorithms and thus similar behavior in their ROC curve outcomes indicating stability toward the interpretation of the results.

In comparison to the WHO endorsed biomarker CRP as a screening test, the best-performing single biomarker, I-309 (AUC 0.87 for logistic regression) alone, outperformed CRP (AUC 0.72 for logistic regression) in our analysis for the whole data set. At a specificity of 70%, I-309 showed 81% sensitivity for logistic regression, whereas CRP obtained only 75% sensitivity. Considering the better performing support vector machines algorithm, the difference was 89% sensitivity for I-309 compared to 74% for CRP. Detailed ROC plots for CRP only are presented in the supplementary material in Fig. S1.

The combination of I-309 with other biomarkers might present technical challenges given its substantially lower absolute concentration (see Table S1 in the Appendix); therefore, we explored the diagnostic performances of signatures that exclude I-309. In this analysis, SYWC showed up as the best-performing single biomarker (AUC 0.86 for logistic regression), closely followed by MIG (AUC 0.83 for logistic regression) and IP-10 (AUC 0.79 for logistic regression).

For biomarker combinations excluding I-309, the two-biomarker combination of SYWC and kallistatin was the most promising (AUC 0.88 for logistic regression). Combining more than three biomarkers did not substantially improve accuracy of the algorithmic performance toward the TPP (see Appendix Fig. S2).

### Stratification by country and HIV status

When assessing the data by country, the single biomarkers and all of their combinations performed substantially worse in the participants from Vietnam than in the other two countries (Fig. 3A through C). When excluding Vietnam, the minimal requirements of the TPP were reached for all 12 biomarkers combined with all the machine learning algorithms except Naïve Bayes, as seen in the ROC plots for the remaining data set (AUC 0.92 with logistic regression excluding Vietnam vs 0.89 with all data combined Fig. 3D and E). Furthermore, the top-performing markers identified at the global level (I-309 and SYWC) were among the best performing markers at country level in South Africa and Vietnam. In Peru, SYWC was followed by CRP and I-309. For the other biomarker combinations, there are great overlaps of the global level with each country level: SYWC was always included in the best-performing two-marker signature for every country, and SYWC and I-309 appeared in the every best performing three-marker signature. See Table 1, Fig. 3, Table S6 in the supplementary material for details on the stratification by country.

For HIV-positive patients, the combination of SYWC and I-309 reached minimal target accuracy of the TPP using SVM and Random Forest (AUC 0.90 and 0.85, respectively); however, the sample size was small (110). No other biomarker subset performed substantially better in HIV-positive or -negative patients. The stratification by HIV status revealed that with three or more biomarkers performance did not improve in both stratification groups. When excluding I-309, the stratification by HIV status leads to similar results as in the full data set.

## DISCUSSION

In this multi-center retrospective nested cohort study, several host biomarkers alone and a combination of two to three biomarkers were substantially better than CRP alone, the screening test currently recommended by WHO. A signature of host biomarkers (I-309, SYWC, and kallistatin) met the minimum WHO criteria for a triage test in adults at the global level (combination of three countries) and at the country level for two of them (Peru and South Africa) and served as the most promising signature to identify active TB disease. These top-performing biomarkers are part of three different signaling pathways, with a complementary nature. While I-309 stimulates chemotaxis of monocytes and is secreted by activated T lymphocytes (30 – 32), SYWC is a gamma interferon-inducible Trp-tRNA-synthetase associated with stress response (33) and kallistatin is an endogenous human serine proteinase inhibitor, that is able to inhibit tissue kallikrein kininogenase and amidolytic activities *in vitro* (34). It is worthwhile noting that kallistatin concentration goes in the opposite direction compared to SYWC and I-309; I-309 and SYWC are upregulated in TB patients, while kallistatin is downregulated. When combining an upregulated marker and downregulated marker in a model, this allows for self-normalization to control for both pre-analytical and analytical sources of variation under the assumption that both proteins undergo the same effects of pre-analytical and analytical variation in the same sample. This also allows for improved signal-to-noise ratios (35). This concept has been applied in other commercial tests (36). No other three-protein marker signature for pulmonary TB in adults was identified in the literature, but for example, a site-independent five-marker signature (37). While ApoA1 performs poorly on its own, it has shown to bring great additional value in combination with other biomarkers, as ApoA1 evolves its unique features mainly in combination with other biomarkers by spanning a greater discriminatory range.

The results were confirmed by different bioinformatics approaches and the most significant and robust biomarkers were identified across different machine learning algorithms. We also investigated simpler models on diagnostic accuracy, like radiometric approaches that divide the concentration of one marker by the concentration of a second marker, which did not lead to performance improvements. This analysis aimed at providing a potential set of host markers that could serve as a diagnostic tool to discern TB and inform a future research agenda towards the development of a host protein biomarker TB triage test. The study benefitted from prior work that screened a large number of possible markers and helped to select the most promising (8, 18 – 20) ones.

This lends hope to the feasibility of a simple low-cost, blood-based host protein biomarker assay as currently available multiplex point-of-care assays for antigens and cardiac biomarkers do include three targets, with the latter often being quantitative. In the context of a TB biomarker signature, the assay would need to be quantitative, preferably present a large dynamic range given the concentration of the biomarker candidates and be linked with some processing unit to compute and display the associated TB call, all these challenges could be addressed by recent fluorescent multiplex immunoassays that run on small and portable devices. Whether such an assay is possible under highly constraining operating conditions (e.g., high temperature and humidity), compatible with streamlined sample preparation requiring serum dilution, and at the <2 USD target ex-works price at scale remains to be proven (6). Defining a cutoff for a globally applicable test might be difficult, given the variability between regions as seen in our data.

The decreased performance of the signature in Vietnam was notable, and could relate to the patient population enrolled (despite inclusion criteria being the same), other concomitant infections, the host immune system, the circulating *Mycobacterium tuberculosis* lineages, or to pre-analytical or storage issues of the sample (38, 39). Differences in the population enrolled are suggested by a larger percentage of smear-negative TB. The impact of the host on acute phase reactants was suggested by others, with the Asian ethnicity being associated with lower median baseline pre-treatment CRP (40). This was also confirmed in our data by CRP response being substantially lower in the Vietnam subset. The pre-analytical factors and storage issues appear less likely as proteins are expected to be largely stable, samples were recently collected and pre-analytical steps were standardized. However, the host and pathogen variability or an interaction of the two could be explanatory. More data are necessary to validate this finding. If this is indeed due to host factors, then such host marker-based tests are necessary to serve a regional market, which is possible but given the low margin on TB diagnostic tests even less attractive for commercialization.

The small sample size of the HIV-positive group in the data set does not allow for general conclusions, in particular, further stratification by country was not possible. Furthermore, an evaluation of these biomarkers in children would be useful to assess the added value in this population, where overall diagnostic capabilities are limited and a test on an easily accessible sample (e.g., blood from a finger prick) is urgently needed.

Our study has several limitations that are noteworthy. First of all, we are aiming to define biomarker combinations suitable for a possible triage test; however, the population used (facility-based), was not representative of a population that would be reached with a community-based triage test, as prevalence was very high. It is very likely that in patients presenting earlier in the disease the performance of an algorithm would be worse. Second, our population was limited in that it did not include regions with high prevalence of other parasitic diseases and additional host genome variability as would be expected in sites for example in South Asia and equatorial Africa. Third, while we were trying to utilize standardized samples from the FIND biobank, possible pre-analytical or storage issues only affecting one site (e.g., Vietnam) could also potentially explain variability observed in the results. Fourth, excluding patients with unclear microbiological results from our sputum-based reference standard could have led to bias in the performance assessment of our non-sputum-based blood signatures. The results of this study may serve as the basis for development of a point-of-care test assay based on a parsimonious protein signature that will require separate validation at chosen cutoffs and for generalizability to other countries. In further work, individuals with non-tuberculous mycobacteria infections may serve as a control group provided that the sample size is sufficiently large.

In conclusion, a host protein biomarker assay is feasible in adults for certain geographical regions based on one, two, or three biomarkers with a performance that meets minimal WHO TPP criteria (i.e., single marker I-309; or combination of I-309, SYWC, and kallistatin to leverage the benefits for assay development outlined above; or I-309 and CRP to leverage existing recommendations). However, more work is needed to validate the results on an independent data set, demonstrate that such as assay can be translated into a practical point-of-care test, and to better understand how to address regional differences in biomarker levels and responses.

## SUPPLEMENTAL MATERIAL

Supplemental figures and tables jcm.00264-23-s0001.pdf This contains supplemental Figures, Tables, as well as additional information regarding MSD U-PLEX assay testing.

ASM does not own the copyrights to Supplemental Material that may be linked to, or accessed through, an article. The authors have granted ASM a non-exclusive, world-wide license to publish the Supplemental Material files. Please contact the corresponding author directly for reuse.
