# Supplementary material for: Diagnostic performance of host protein signatures as a triage test for active pulmonary TB
Source: J Clin Microbiol. 2023 Sep 19;61(10):e00264-23. doi: 10.1128/jcm.00264-23 (PMC10654108; doi:10.1128/jcm.00264-23)
Supplement: Supplemental figures and tables — This contains supplemental Figures, Tables, as well as additional information regarding MSD U-PLEX assay testing. [file jcm.00264-23-s0001.pdf]

Supplementary Material to:

Diagnostic performance of host protein signatures as a triage test for active pulmonary TB

Lisa Koeppel[1*], Claudia M Denkinger[1,2,7], Romain Wyss[2], Tobias Broger[1,2], Novel N Chegou[8], Jill M Dunty[3], Kerry Scott[3], Tatiana Cáceres[4], Elloise Dutoit[5], Cesar Ugarte-Gil[4,11], Mark Nicol[5,6], Eduardo Gotuzzo[4], Paul L A M Corstjens[9], Annemieke Geluk[10], Jayne Sutherland[12], George B Sigal[3], Emmanuel Moreau[2], Audrey Albertini[2], Anna Mantsoki[2], Stefano Ongarello[2], Gerhard Walzl[8], Marta Fernandez Suarez[2]

[1]Division of Infectious disease and Tropical Medicine, University of Heidelberg, Heidelberg, Germany

[2]FIND, Geneva, Switzerland

[3]Meso Scale Diagnostics, LLC., Rockville, Maryland, United States of America

[4]Instituto de Medicina Tropical Alexander von Humboldt, Universidad Peruana Cayetano Heredia, Lima, Perú

[5]Division of Medical Microbiology at the University of Cape Town (UCT), Cape Town, South Africa

[6]Division of Infection and Immunity, School of Biomedical Sciences, University of Western Australia, Perth, Australia

[7]German Center for Infection Research (DZIF), Heidelberg University Hospital Partner Site, Heidelberg, Germany

[8]DSI-NRF Centre of Excellence for Biomedical Tuberculosis Research; South African Medical Research Council Centre for Tuberculosis Research; Division of Molecular Biology and Human Genetics, Faculty of Medicine and Health Sciences, Stellenbosch University, Cape Town, South Africa

[9]Department of Cell and Chemical Biology, Leiden University Medical Center, Leiden, Netherlands

[10]Department of Infectious Diseases, Leiden University Medical Center, Leiden, The Netherlands

[11]School of Medicine, Universidad Peruana Cayetano Heredia (UPCH), Lima, Peru

[12]TB Research Group, Vaccines and Immunity Theme, MRC Unit The Gambia at LSHTM, Fajara, The Gambia.

*Corresponding author Email: Lisa Koeppel, l.koeppel@uni-heidelberg.de

Figure S1: Performance of ApoA1 and CRP individually

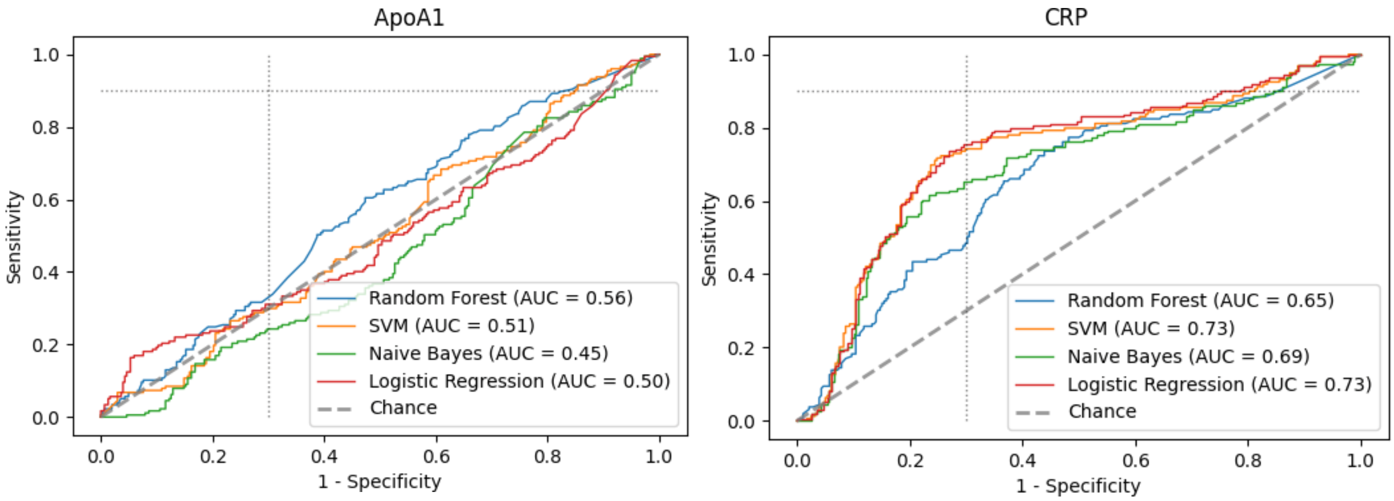

Caption: The dotted line indicates the TPP with 90% sensitivity and 70% specificity.

Abbreviations: SVM: Support Vector Machine; AUC: area under the curve

Figure S2: When excluding I-309, taking more than 3 biomarkers did not substantially improve accuracy of the prediction towards the TPP.

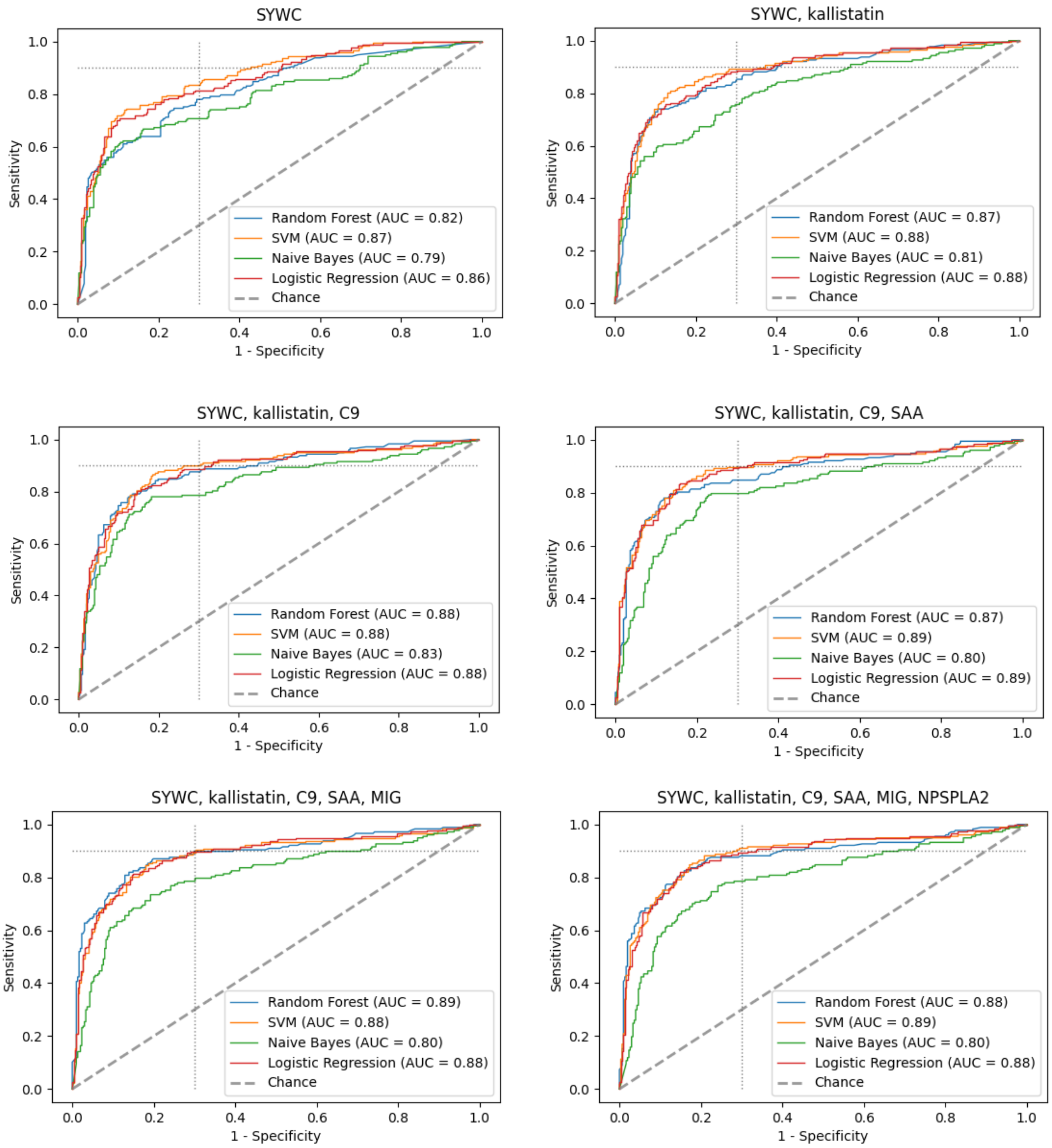

Figure S3: Most promising biomarker combination for HIV positive patients reaching minimal target accuracy of the TPP with all algorithms except Naïve Bayes. The sample size is 110.

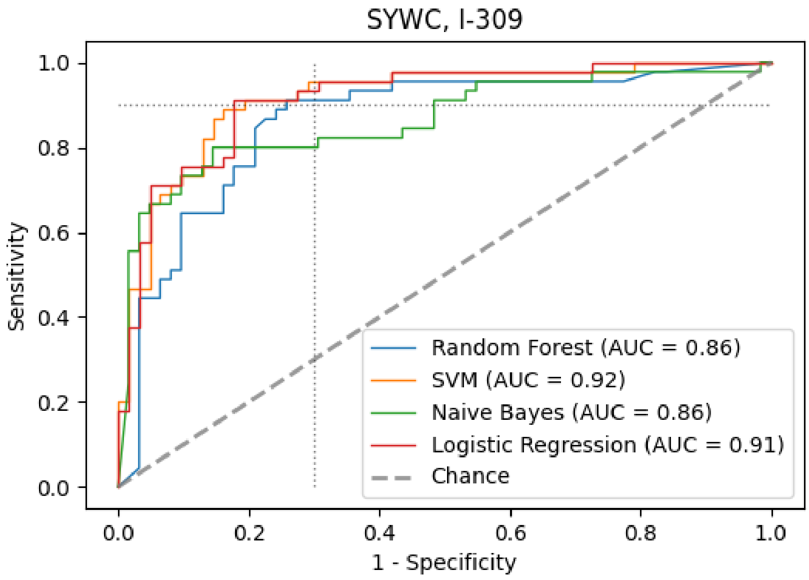

Table S1: Quantitative data on concentration and fold-changes of the single host markers at the global level

Host marker	Quantative value<br>TB positives<br>Mean, [25th and 75th percentiles]	Quantative value<br>TB negative<br>Mean, [25th and 75th percentiles]	Fold change<br>TB negative/<br>TB positive<br>Mean, [25th and 75th percentiles]
NPS-PLA2	244205<br>[23340, 262547]	100354<br>[10250, 38946]	0.41<br>[0.44, 0.15]
SYWC	201565<br>[74824, 245329]	60320<br>[32178, 67047]	0.3<br>[0.43, 0.27]
C9	87393926<br>[58647252, 104369085]	58914995<br>[34953155, 69637726]	0.67<br>[0.6, 0.67]
LBP	10638841<br>[4960943, 12109847]	5779719<br>[2566144, 6192901]	0.54<br>[0.52, 0.51]
CRP	184468404<br>[44442307, 260598048]	67569950<br>[2747640, 44603133]	0.37<br>[0.06, 0.17]
SAA	270013188<br>[22332527, 301088370]	131027361<br>[2291012, 29324382]	0.49<br>[0.1, 0.1]
Kallistatin	16858488<br>[10120463, 20992742]	26393738<br>[17787049, 30075939]	1.57<br>[1.76, 1.43]
Ferritin	640926<br>[175864, 735009]	342358<br>[72881, 416977]	0.53<br>[0.41, 0.57]
ApoA1	2137295290<br>[626983785, 1542702584]	10841296498<br>[977186634, 1921571968]	5.07<br>[1.56, 1.25]
IP-10	1958<br>[487, 2348]	571<br>[173, 599]	0.29<br>[0.36, 0.25]
I-309	126<br>[36, 100]	24<br>[15, 27]	0.19<br>[0.41, 0.27]
MIG	6266<br>[2082, 6809]	1558<br>[585, 1704]	0.25<br>[0.28, 0.25]

Table S2: Quantitative data on concentration and fold-changes of the single host markers in Peru for the top 3 performing host markers

Host marker Ranking	Quantative value TB positives Mean, [25th and 75th percentiles]	Quantative value TB negative Mean, [25th and 75th percentiles]	Fold change TB negative/ TB positive Mean, [25th and 75th percentiles]
Best: SYWC	165617, [75509, 210373]	32060, [21220, 31858]	0.19 [0.28, 0.15]
2nd best: CRP	215182996, [69348432, 289583579]	13990406, [2853470, 13741375]	0.07 [0.04, 0.05]
3rd best: I-309	86, [47, 92]	22, [13, 25]	0.26, [0.29, 0.27]

Table S3: Quantitative data on concentration and fold-changes of the single host markers in

South Africa for the top 3 performing host markers

Host marker Ranking	Quantative value TB positives Mean, [25th and 75th percentiles]	Quantative value TB negative Mean, [25th and 75th percentiles]	Fold change TB negative/ TB positive Mean, [25th and 75th percentiles]
Best: I-309	177, [47, 162]	23, [14, 24]	0.13, [0.3, 0.15]
2nd best: SYWC	248059, [137744, 308120]	77205, [44388, 89431]	0.31, [0.32, 0.29]
3rd best: MIG	6800, [3133, 8563]	1626, [439, 1934]	0.24, [0.14, 0.23]

Table S4: Quantitative data on concentration and fold-changes of the single host markers in Vietnam for the top 3 performing host markers

Host marker	Quantative value<br>TB positives<br>Mean, [25th and 75th percentiles]	Quantative value<br>TB negative<br>Mean, [25th and 75th percentiles]	Fold change<br>TB negative/<br>TB positive<br>Mean, [25th and 75th percentiles]
Best: SYWC	249225,<br>[68864, 273974]	65683,<br>[41459, 72606]	0.26,<br>[0.6, 0.27]
2nd best: I-309	135,<br>[34, 75]	27,<br>[17, 31]	0.2<br>[0.5, 0.42]
3rd best: IP-10	2052,<br>[500, 2020]	644,<br>[336, 647]	0.31<br>[0.67, 0.32]

Table S5: Definition of TB status

Description	TB status
Positive MTB culture	Definite tuberculosis<br>(smear pos / smear neg)
Negative MTB culture, negative smear and response to TB treatment	Clinical tuberculosis
Smear negative, Xpert and culture negative on all sputum samples and exhibition symptom resolution in the absence of tuberculosis treatment at the 2–3-month follow-up visit	Non-tuberculosis disease

Table S6: Biomarker combinations ranked according to their value of the negative loglikelihood stratified by country

No of biomarkers combined	**Best combination** [marker(s)] Loglikelihood, AUC for logistic regression	**Second best combination** [marker(s)] Loglikelihood, AUC for logistic regression	**Third best combination** [marker(s)] Loglikelihood, AUC for logistic regression
South Africa			
1	[I-309] 60, 0.89	[SYWC] 66, 0.90	[MIG] 73, 0.89
2	[SYWC, I-309] 51, 0.93	[C9, I-309] 52, 0.90	[kallistatin, I-309] 55, 0.89
3	[SYWC, I-309, C9] 47, 0.93	[I-309, C9, SAA] 48, 0.91	[I-309, SYWC, SAA] 50, 0.93
Peru			
1	[SYWC] 26, 0.88	[CRP] 29, 0.88	[I-309] 29, 0.90
2	[SYWC, C9] 40.1, 0.92	[ApoA1, CRP] 40.5, 0.88	[I-309, SYWC] 40.6, 0.92
3	[SYWC, I-309, ApoA1] 19, 0.93	[SYWC, CRP, ApoA1] 19, 0.91	[SYWC, C9, ApoA1] 20, 0.92
Vietnam			
1	[SYWC] 74, 0.84	[I-309] 82, 0.82	[IP-10] 89, 0.76
2	[SYWC, Ferritin] 73, 0.84	[SYWC, I-309] 74, 0.84	[SYWC, kallistatin] 74, 0.83
3	[SYWC, I-309, Ferritin] 72, 0.84	[SYWC, Ferritin, kallistatin] 73, 0.83	[SYWC, kallistatin, SAA] 73, 0.83

Text S1: Additional information regarding MSD U-PLEX assay testing

For each host biomarker, antibodies were first screened to identify suitable antibody pairs when not previously identified by MSD (kallistatin, NPS-PLA2, SYWC, ferritin) and single-plex testing was conducted for calibrator titration and native protein recognition in human serum. Then, feasibility of multiplexing and further qualification of the panels were performed by

collecting data on cross-reactivity between the members of the panel, calibrator curves, dilution linearity in diluent, spike recovery in diluent, reproducibility and finally stability of the host biomarkers in human serum.

Each plate run during sample testing included a set of eight calibration samples (created by a 1:4 serial dilution of the assay calibration standard), the three controls and up to 37 serum samples, all run in duplicate. The assays were calibrated by fitting the relationship of the calibration sample signals to their assigned concentrations with a four-parameter logistic (4-PL) model using $1/Y^2$ weighting. Quantitation of the samples and controls was carried out by back-fitting the assay signals to the 4-PL model, and then correcting for the sample dilution.

The custom assay panels were created using antibodies pairs that are available commercially from MSD as R-PLEX® or U-PLEX antibody sets, with the exception of the NPS-PLA2 and SYWC assays, which used prototype antibody pairs selected specifically for this study. The custom panels were qualified through verification studies characterizing cross-reactivity between the assay targets in each panel, dilution linearity in diluent, spike recovery in diluent, reproducibility and stability of the host biomarkers in human serum.
